# PKHB1 peptide induces antiviral effects through induction of immunogenic cell death in herpes simplex keratitis

**DOI:** 10.3389/fphar.2022.1048978

**Published:** 2022-12-01

**Authors:** Yun He, Chenchen Wang, Qi Liang, Rongjie Guo, Jiaxuan Jiang, Wenhao Shen, Kai Hu

**Affiliations:** Department of Ophthalmology, Affiliated Drum Tower Hospital, Medical School of Nanjing University, Nanjing, China

**Keywords:** herpes simplex keratitis (HSK), herpes simplex virus type 1 (HSV-1), PKHB1 peptide, immunogenic cell death (ICD), antigen-presenting cells (APCs)

## Abstract

Herpes simplex keratitis (HSK) is a severe, infectious corneal disease caused by herpes simplex virus type 1 (HSV-1) infection. The increasing prevalence of acyclovir resistance, the side effects of hormonal drugs, and the ease of recurrence after surgery have made it crucial to develop new methods of treating HSK. HSV-1 evades the host immune response through various mechanisms. Therefore, we explored the role of the immunogenic cell death inducer PKHB1 peptide in HSK. After subconjunctival injection of PKHB1 peptide, we observed the ocular surface lesions and survival of HSK mice and detected the virus levels in tear fluid, corneas, and trigeminal ganglions. We found that PKHB1 peptide reduced HSV-1 levels in the eye and alleviated the severity of HSK. Moreover, it increased the number of corneal infiltrating antigen-presenting cells (APCs), such as macrophages and dendritic cells, and CD8^+^ T cells in ocular draining lymph nodes. We further observed that PKHB1 peptide promoted the exposure of calreticulin, as well as the release of ATP and high-mobility group box 1 in HSV-1-infected cells *in vitro*. Our findings suggested that PKHB1 peptide promoted the recruitment and maturation of APCs by inducing the release of large amounts of damage-associated molecular patterns from infected cells. APCs then phagocytized antigenic materials and translocated to the lymph nodes, triggering a cytotoxic T lymphocyte-dependent immune response that ultimately alleviated HSK.

## Introduction

Herpes simplex virus type 1 (HSV-1) is a double-stranded deoxyribonucleic acid virus. HSV-1 infections are widespread, with 67% of the global population reported to have been exposed to it (Tognarelli et al., 2019). Herpes simplex keratitis (HSK) is a severe infectious corneal disease caused by HSV-1 infection, which is the primary cause of corneal blindness (Jamali et al., 2020; Chen et al., 2021). The current treatment for HSK is mainly antiviral eye drops and topical application of glucocorticoids. Antiviral therapy is limited to suppressing viral replication and is ineffective against latent viruses. Studies have also reported viral resistance to acyclovir (Frobert et al., 2014; Rousseau et al., 2017). Topical corticosteroid therapy can lead to numerous sequelae, including corneal scarring, neovascularization, and neurotrophic keratitis, and is also associated with steroid-responsive glaucoma and cataract formation (Wilhelmus et al., 1996). Therefore, there is an urgent need to develop new treatments.

After the invasion of HSV-1 into the host, the immune response generated by the host struggle with the immune escape caused by the pathogen. Macrophages directly phagocytize the viruses and secrete various cytokines to inhibit HSV-1 replication (Kim et al., 2013). Dendritic cells (DCs) act as antigen-presenting cells (APCs) to present HSV-1 antigen to T lymphocytes, which activate the immune response of T lymphocytes (Tognarelli et al., 2019). Cytotoxic T lymphocytes inhibit HSV-1 by releasing granzyme and interferon-γ (IFN-γ) (Rowe et al., 2013). HSV-1 can evade the host immune response through various mechanisms. HSV-1 protein infected-cell protein 0 (ICP0) promotes the degradation of CD83 costimulatory molecule, which impacts the antigen-presentation capacity of DCs (Kummer et al., 2007; Heilingloh et al., 2014; Grosche et al., 2020). Viruses reduce their migratory ability toward CCL19 and CXCL12 chemokine by reducing CCR7 and CXCR4 expression (Prechtel et al., 2005). HSV-1 infected cell protein 47 (ICP47) has been extensively reported to inhibit MHC antigen presentation by interacting with the transporter associated with antigen presentation and consequently attenuate virus-specific cytotoxic T lymphocytes (CTLs) responses (Wang et al., 2013). Multiple mechanisms exist by which HSV-1 can subvert host immune responses and cellular machinery to promote virus propagation and persistence (Su et al., 2016). So, developing new therapies that stimulate the immune system is crucial for treating HSK.

Immunogenic cell death (ICD) belongs to regulated cell death, a form of cell death that enhances the organismal immunity in an immunocompetent host and can counteract immune escape caused by tumors or infections (Galluzzi et al., 2020). ICD is characterized by the exposure or release of endogenous immunogenic biological factors, i.e., damage-associated molecular patterns (DAMPs), including calreticulin (CALR) (Obeid et al., 2007; Garg et al., 2012; Fucikova et al., 2018), heat shock proteins 70 and 90 (HSP70 and HSP90) (Spisek et al., 2007; Garg et al., 2015), ATP (Elliott et al., 2009; Aymeric et al., 2010), and the non-histone chromatin protein high-mobility group box 1 (HMGB1) (Scaffidi et al., 2002; Inoue and Tani, 2014). These DAMPs recruit APCs to ICD sites and stimulate the uptake, processing, and presentation of dead cell-associated antigens, resulting in the adaptive immune response (Tesniere et al., 2008; Krysko et al., 2012; Bezu et al., 2015).

Calvillo-Rodríguez et al. (2022) recently designed a peptide named PKHB1 based on the structure-activity relationship of the C-terminal binding domain (CBD) of platelet response protein-1 (TSP-1). The peptide sequence is kRFYVVMWKk with terminal D-lysines (Denèfle et al., 2019; Leclair et al., 2020). PKHB1 is a peptide mimetic that is stable in mouse and human serum and can induce cell death involving CD47 activation in different cancer cells, especially in hematologic malignancies and breast cancer. In breast cancer cells, PKHB1 peptide induces mitochondrial alterations, ROS production and calcium-dependent cell death (Calvillo-Rodríguez et al., 2022). In leukemic cells, PKHB1 peptide induces CALR exposure and DAMPs release leading to ICD *in vitro*, and prophylactic vaccination inhibits tumor formation *in vivo* (Uscanga-Palomeque et al., 2019). This peptide is currently being investigated in preclinical studies for CLL (χ-Pharma) (Martínez-Torres et al., 2019; Uscanga-Palomeque et al., 2019). However, little is known about antiviral effects and the cell death capacity of PKHB1 peptide in infectious diseases. Our current study investigated whether PKHB1 peptide could be used to treat HSK and the underlying mechanisms. We first evaluated the effect of PKHB1 peptide on clinical signs and ocular tissue viral level expression in HSK mice. Furthermore, since intrinsic immune cells are the first line of defense against HSV-1 infection, we explored the effect of PKHB1 peptide on corneal macrophages and DCs. We further explored the changes of T cells in the draining lymph nodes (dLNs). Finally, we analyzed that PKHB1 peptide plays a role as an ICD inducer in HSK disease *in vitro*.

## Materials and methods

### Cells and viruses

Vero cells were a gift from Professor Wu Zhiwei of Nanjing University. Vero cells, Mouse leukemia cells of monocyte macrophage line (RAW264.7, CoBioer Biosciences Co., Ltd. Nanjing, China) and Human corneal endothelial cell line (B4G12, DSMZ; ACC-647, Shanghai GuanDao Biological Engineering Co., Ltd.) were cultured in Dulbecco’s modified Eagle medium (DMEM) high glucose medium (Gibco, United States), Human corneal epithelial cell line (HCEC, CRL-11135, HCE-2; ATCC, Manassas, VA) were cultured in DMEM/F12 medium (Gibco, United States), and human myeloid leukemia mononuclear cell line (THP-1, CoBioer Biosciences Co., Ltd. Nanjing, China) were cultured in RPMI Medium 1640 (Invitrogen, Carlsbad, CA, United States), with 10% (v/v) fetal calf serum, 100 U/ml penicillin, and 100 g/ml streptomycin (Gibco, United States) in a humidified 37°C 5% CO_2_ incubator. HSV-1 strain McKrae was kindly provided by Dr. Pedram Hamrah of Schepens Eye Research Institute, Department of Ophthalmology, Harvard Medical School, Boston, MA, United States, which was prepared as previously described (Jamali et al., 2020; Liu et al., 2021). Viruses were propagated, and titers were determined by plaque assay on Vero cells (Knipe and Spang, 1982).

### Peptide synthesis

The peptide sequence is kRFYVVMWKk. PKHB1 peptide was synthesized and purified by Genscript (Nanjing, China) and dissolved in PBS at different concentrations. The molecular weight of the peptide was measured by mass spectrometry, and the purity of the peptide was demonstrated by high-performance liquid chromatography.

### Animals and herpes simplex keratitis mouse model

All animal experiments were approved by the Animal Research Ethics Committee of Nanjing Drum Tower Hospital and were conducted under the guidance of the Laboratory Animal Management Committee of Jiangsu Province, and in accordance with the relevant regulations established by the Vision and Ophthalmology Research Society. The experimental animals were C57BL/6 male mice at about 6 weeks of age with normal eye development, all purchased from the Animal Center of Nanjing Medical University and kept at the SPF level in the Animal Experiment Center of Nanjing Drum Tower Hospital (Nanjing, China). The feeding environment temperature was about 25°C, and humidity was kept between 40% and 50%. All mice were anesthetized by intraperitoneal injection of 1% sodium pentobarbital (80 mg/kg), and the eyes were inoculated with 5 µl of HSV-1 strain McKrae (1 × 10^6^ PFU/ml) by 5 (horizontal) × 5 (vertical) strokes on the right cornea with a 30-gauge needle. The infection was conducted unilaterally. 5 µl of PKHB1 peptide at a concentration of 200 µM was injected subconjunctivally on the day before infection, the first day after infection and the second day after infection, respectively, to observe its action *in vivo*. Eyes of the mice were observed, tear samples were taken with eye swabs to measure the virus titers, and tissues were obtained for the experiments at 3 days post-infection (d p. i.) and 6 d p.i..

### Subconjunctival injection

Before injection, we administered a drop of oxybuprocaine hydrochloride in the eye of the mice for topical anesthesia. An insulin syringe with a 30G needle was used for subconjunctival injection. The conjunctiva was gently pulled from the sclera with a pair of forceps. PKHB1 peptide was injected into the eye’s subconjunctival area (5 µl) under a body microscope. The injection site was located at the junction between the iris and conjunctiva, and the needle was placed in the horizontal direction at 10°–15° to the ocular surface. Successful injection of a blister into the conjunctiva bulge was observed. After injection, we instilled one drop of ciprofloxacin.

### Cytotoxicity assay

Different cells including HCECs, B4G12 cells, RAW264.7 cells, and THP1 cells, were seeded in 96-well plates separately. The supernatants were removed, and a new medium with increasing doses of PKHB1 peptide was added. After 24 h, the medium was replaced with 100 µl of fresh medium containing 10 µl of Cell Counting Kit 8 (CCK-8) reagent (Vazyme, Nanjing, China) for 2 h. MRX II microplate reader (Dynex, Chantilly, VA, United States) was used to measure OD values for each well at 450 nm. Cell viability values for treated cells were normalized with those of untreated cells.

### Herpes simplex keratitis lesion extent score and sodium fluorescein staining score of the cornea

The corneal HSK lesions of the infected mice were observed by the body microscope and scored according to the following criteria:

0 point: no epithelial lesions or punctate lesions, no edema or cloudiness of the stroma;
1 point: stellate epithelial lesions or mild edema and cloudiness of the stroma;
2 points: dendritic or atlas-like epithelial lesions covering less than 25% of the cornea, stromal edema or cloudy lesions less than one-half of the corneal diameter;
3 points: dendritic or atlas-like epithelial lesions covering 25%–50% of the corneal area, stromal edema or cloudy lesions larger than one-half of the corneal diameter;
4 points: dendritic or atlas-like epithelial lesions occupying >50% of the corneal area, severe stromal edema and clouding, with no visible iris.

A small amount of 1% sodium fluorescein stain (Jingming, Tianjin, China) was dipped on the conjunctiva of the inferior fornix of the mice. Cobalt blue light was used to observe the lesions stained yellow-green on the cornea. The corneal fluorescein score (CFS) was assessed using a masked approach consistent with the National Eye Institute standard scoring system (0–3 points for each of the four regions of the cornea).

### Hematoxylin-eosin staining of tissue sections

The mice were sacrificed at 3 d p.i. and 6 d p.i.. Eyeballs were immersed in 4% paraformaldehyde for 24 h to be fixed and embedded in paraffin after dehydration with an ethanol gradient (70, 80, 90, 95, and 100%). The eyeballs tissue was sliced into 3-μM-thick sections, deparaffinized with xylene, and then hydrated in an ethanol gradient. Then, the sections were stained with hematoxylin-eosin staining kit (Servicebio, Wuhan, China). Photos were taken of the sections using a light microscope (Leica Microsystems, Wetzlar, Germany).

### Determination of 50% tissue culture infectious dose

Tear samples were taken at 3 d p.i. and 6 d p.i.. The eyes of the mice were kept open during this process. 10 µl of sterile PBS were administered as eye drops and retained in the ocular area for 1 min, and then the liquid was aspirated with an eye swab without touching the cornea. The whole process follows sterile operation protocols. Vero cells were seeded in 96-well plates. The supernatants were removed, a new medium of DMEM containing 2% FBS was added, and the cell culture was maintained for 24 h. Then, 10-fold serial dilutions of HSK mice tear samples were used to infect monolayer Vero cells for 72 h. Eight replicates were made for each dilution. The number of wells in which cytopathic effects appeared for each dilution gradient was counted under a light microscope, and the percentages were calculated. Calculations of virus titers were performed according to the Reed-Muench method (Capelli et al., 2020).

### RNA isolation and quantitative real-time polymerase chain reaction

Total RNA was extracted from cells and mice tissues using TRIzol reagent (Takara, Japan). The cDNA was compounded by reverse transcription of 1 µg of total RNA using the HiScript II Q Select RT SuperMix (Vazyme, Nanjing, China) according to the manufacturer’s instructions. The ChamQ Universal SYBR qPCR Kit (Vazyme, Nanjing, China) was used in performing RT-PCR on ABI QuantStudio six Flex (Invitrogen, Carlsbad, CA, United States). GAPDH was used for the normalization of mRNA, and relative expressions of genes were calculated by the 2^−ΔΔCT^ method. The sequences of primers are listed in Table 1.

**TABLE 1:** Primers used for qRT-PCR.

Gene	Species	Sequence forward (5′ to 3′)	Sequence reverse (5′ to 3′)
GAPDH	Mouse	TGA​TGA​CAT​CAA​GAA​GGT​GGT​GAA​G	TCC​TTG​GAG​GCC​ATG​TGG​GCC​AT
GAPDH	Human	GGA​GCG​AGA​TCC​CTC​CAA​AAT	GGC​TGT​TGT​CAT​ACT​TCT​CAT​GG
gB		AACGCGACGCACATCAAG	CTG​GTA​CGC​GAT​CAG​AAA​GC
INOS	Mouse	GTT​CTC​AGC​CCA​ACA​ATA​CAA​GA	GTG​GAC​GGG​TCG​ATG​TCA​C
CD86	Mouse	TCA​ATG​GGA​CTG​CAT​ATC​TGC​C	GCC​AAA​ATA​CTA​CCA​GCT​CAC​T
TNFα	Mouse	CCC​TCA​CAC​TCA​GAT​CAT​CTT​CT	GCT​ACG​ACG​TGG​GCT​ACA​G
IL-1β	Mouse	GAA​ATG​CCA​CCT​TTT​GAC​AGT​G	TGG​ATG​CTC​TCA​TCA​GGA​CAG
IL-6	Mouse	TAG​TCC​TTC​CTA​CCC​CAA​TTT​CC	TTG​GTC​CTT​AGC​CAC​TCC​TTC
Arg-1	Mouse	CTC​CAA​GCC​AAA​GTC​CTT​AGA​G	GGA​GCT​GTC​ATT​AGG​GAC​ATC​A
CD206	Mouse	CTC​TGT​TCA​GCT​ATT​GGA​CGC	TGG​CAC​TCC​CAA​ACA​TAA​TTT​GA
IL-10	Mouse	GCT​CTT​ACT​GAC​TGG​CAT​GAG	CGC​AGC​TCT​AGG​AGC​ATG​TG
CD11c	Mouse	TCC​CTG​AAC​TCA​CGA​GTC​TTT	GGT​TGT​CAA​GTC​CGT​AAA​ATG​C
IFN-γ	Mouse	ATG​AAC​GCT​ACA​CAC​TGC​ATC	CCA​TCC​TTT​TGC​CAG​TTC​CTC

### Immunofluorescence staining of corneal tissue

Immunofluorescence staining of corneal tissue was performed as described previously (Jamali et al., 2020). The intact mouse corneas were placed in 4% paraformaldehyde, fixed at RT for 30 min, and then placed into 0.5% Triton-X 100 to break the membrane at RT for 30 min. Samples were incubated for 1 h in 5% donkey serum at RT to avoid non-specific staining. Subsequently, corneas were incubated with antibodies against F4/80 (Clone: BM8, Biolegend, Inc. San Diego, CA) or CD11c (Clone: N418, Biolegend, Inc. San Diego, CA) (1:200) overnight at 4°C. The next day, samples were washed and covered with the DAPI-containing medium (Abcam, Cambridge, MA, United States). Staining observation and analysis was performed using the Leica Thunder system (Leica, Wetzlar, Germany) and confocal laser scanning microscope (Olympus BX53, Tokyo, Japan).

### Single cell suspension and flow cytometry analysis

Single-cell suspensions were prepared by grinding mice’s dLNs and filtering them using 70 μm cell filters. The lymph nodes were transferred to filters placed on centrifuge tubes. The lymph nodes were gently ground with the inner core of a 1 ml syringe, and filters were continuously rinsed with PBS until only connective tissue remained. Fixable Viability Stain 780 (BD, San Jose, CA) was stained for 10 min at RT to the dead live cell assay before proceeding to the other destination antibody experiments. To assess the proportion of various immune cells, single cell suspensions of dLNs were stained with PE-CYANINE7-conjugated anti-CD45 (Clone: 30-F11, 0.65 µl/test, Thermo Fischer Inc. Waltham, MA), FITC-conjugated anti-F4/80 (Clone: BM8, 2 µl/test, Biolegend, Inc. San Diego, CA), APC-Cy™7-conjugated anti-CD11b (Clone: M1/70, 2 µl/test, BD, San Jose, CA), APC-conjugated anti-CD86 (Clone: GL1, 0.3 µl/test, eBioscience, San Diego, CA, United States), FITC-conjugated anti-CD11c (Clone: N418, 0.5 µl/test, Biolegend, Inc. San Diego, CA), PE-conjugated anti-MHC-II (Clone: M5/114.15.2, 0.1 µl/test, eBioscience, San Diego, CA, United States), FITC-conjugated anti-CD4 (Clone: GK1.5, 0.2 µl/test, Biolegend, Inc. San Diego, CA), and APC-conjugated anti-CD8 (Clone: 53–6.7, 0.6 µl/test, eBioscience, San Diego, CA, United States) for 30 min at 4°C. Antibodies diluted by Flow Stain Buffer Soln (eBioscience, San Diego, CA, United States). Experiments included isotype-matched antibodies as controls. They were stained with PE-CYANINE7 Isotype Control (Clone: eB149/10H5, Invitrogen, Carlsbad, CA), FITC Isotype Control (Clone: R35-95, BD, San Jose, CA), APC Isotype Control (Clone: eBR2a, Invitrogen, Carlsbad, CA), PE Isotype Control (Clone: R35-38, BD, San Jose, CA), APC-Cy™7 Isotype Control (Clone: A95-1, BD, San Jose, CA). Flow cytometry analysis was run using BD Accuri C6 (BD, San Jose, CA) and Aria II (BD, San Jose, CA). The data were analyzed by FlowJo V9.2 (FlowJo, LLC, Ashland, OR).

### Calreticulin exposure

HCECs were cultured *in vitro* and infected with HSV-1 (MOI = 1). Infected HCECs in 6-well dishes were treated with PBS or PKHB1 peptide at 100 µM for 3 h. Then, the cells were placed in 4% paraformaldehyde, fixed at RT for 30 min, and then placed into 0.5% Triton-X 100 to break the membrane at RT for 10 min. Cells were incubated for 1 h in 5% donkey serum at RT to avoid non-specific staining. Subsequently, cells were incubated with CALR antibody (1:400, Clone: D3E6, Cell Signaling Technology, Massachusetts, United States) overnight at 4°C. The next day, cells were washed, incubated with fluorescent secondary antibody at 37°C for 1 h, and covered with the DAPI-containing medium (Abcam, Cambridge, MA, United States). Staining observation and analysis was performed using the Leica Thunder system (Leica, Wetzlar, Germany).

### ATP and high-mobility group box 1 release assay

HCECs were cultured *in vitro* and infected with HSV-1 (MOI = 1). Infected HCECs in 6-well dishes were treated with PBS or PKHB1 peptide at 100 µM for 3 h. The supernatants were recovered, centrifuged at 2000 RPM for 10 min, and used to assess extracellular ATP by ATP Assay Kit (Beyotime Institute of Biotechnology, Haimen, China), or HMGB1 using the HMGB1 ELISA kit (Mlbio, Shanghai, China) following the manufacturer’s instructions. MRX II microplate reader (Dynex, Chantilly, VA, United States) was used to measure OD values for each well at 450 nm.

### Statistical analysis

Each method described above involved at least three biological replicates, and all experiments were repeated at least three times. The experimental data were processed with GraphPad Prism 8.0 (GraphPad, San Diego, CA, United States) and statistically analyzed. Statistical differences between groups were analyzed by t-tests, One-way ANOVA or Two-way ANOVA. Statistical significance was indicated by *p* < 0.05.

## Results

### Identification of PKHB1 peptide

**FIGURE 1:**
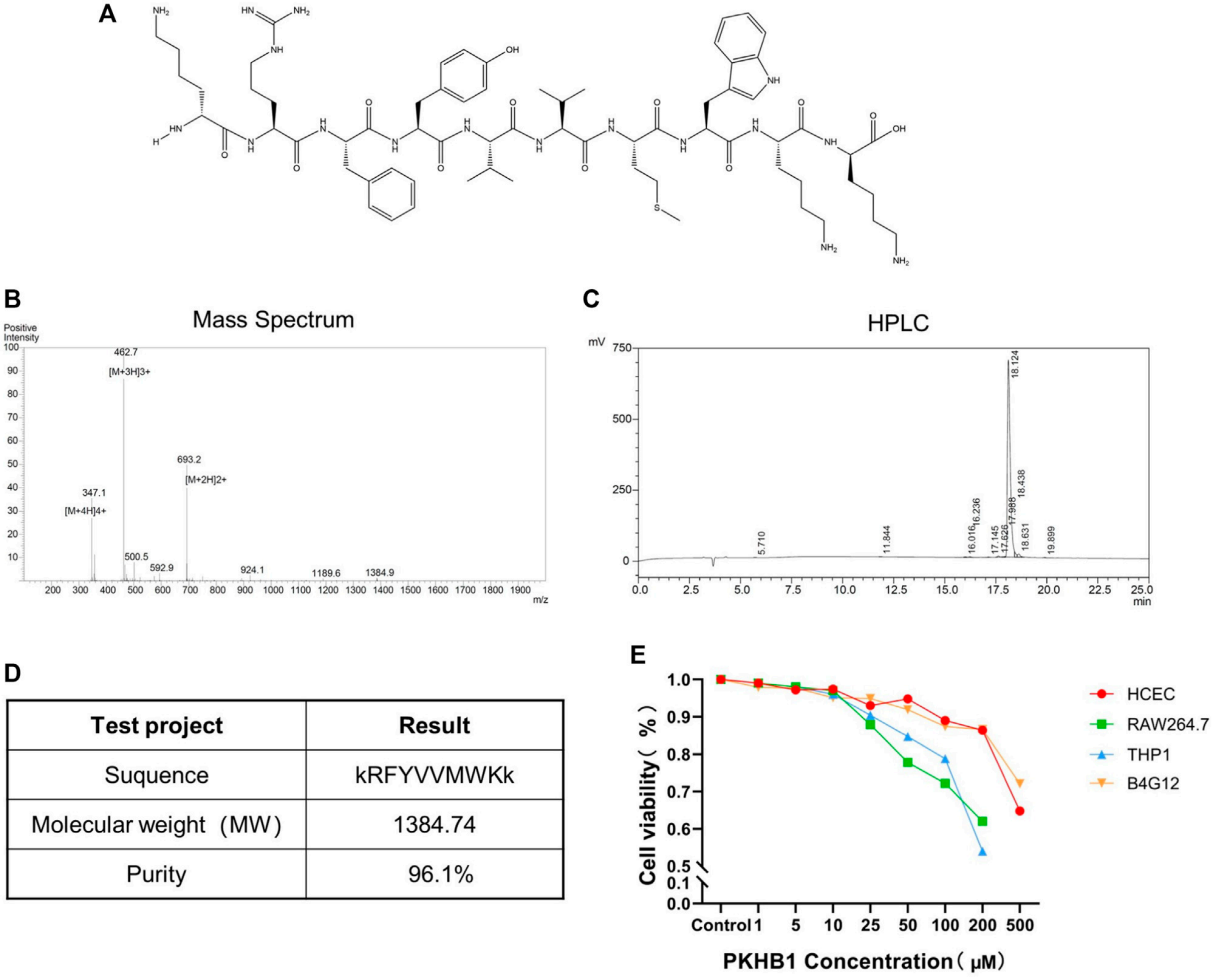
Introduction to PKHB1 peptide. **(A)** The structure of PKHB1 peptide. **(B)** Detection of peptide molecular weight by mass spectrometry. **(C)** High-performance liquid chromatography to identify the purity of peptides. **(D)** Summary table of the above items. **(E)** The cytotoxicity of PKHB1 peptide on HCECs, B4G12 cells, RAW264.7 cells and THP1 cells for 24 h was determined by Cell Counting Kit 8 (CCK-8) assays.

As shown in Figure 1A, the peptide sequence was kRFYVVMWKk with terminal D-lysines. Its relative molecular mass was 1,384.74 determined by mass spectrometry, and its purity was 96.1% determined by high-performance liquid chromatography (Figures 1B–D). The cytotoxicity levels of PKHB1 peptide in different cells including HCECs, B4G12 cells, RAW264.7 cells and THP1 cells were detected by CCK8 assay (Figure 1E), and CC_50_ values of different cells were greater than 200 μM. Therefore, we chose PKHB1 peptide with a concentration of 200 μM for subsequent experiments *in vivo*.

### PKHB1 peptide inhibited herpes simplex virus type 1 replication and reduced herpes simplex keratitis disease severity *in vivo*

**FIGURE 2:**
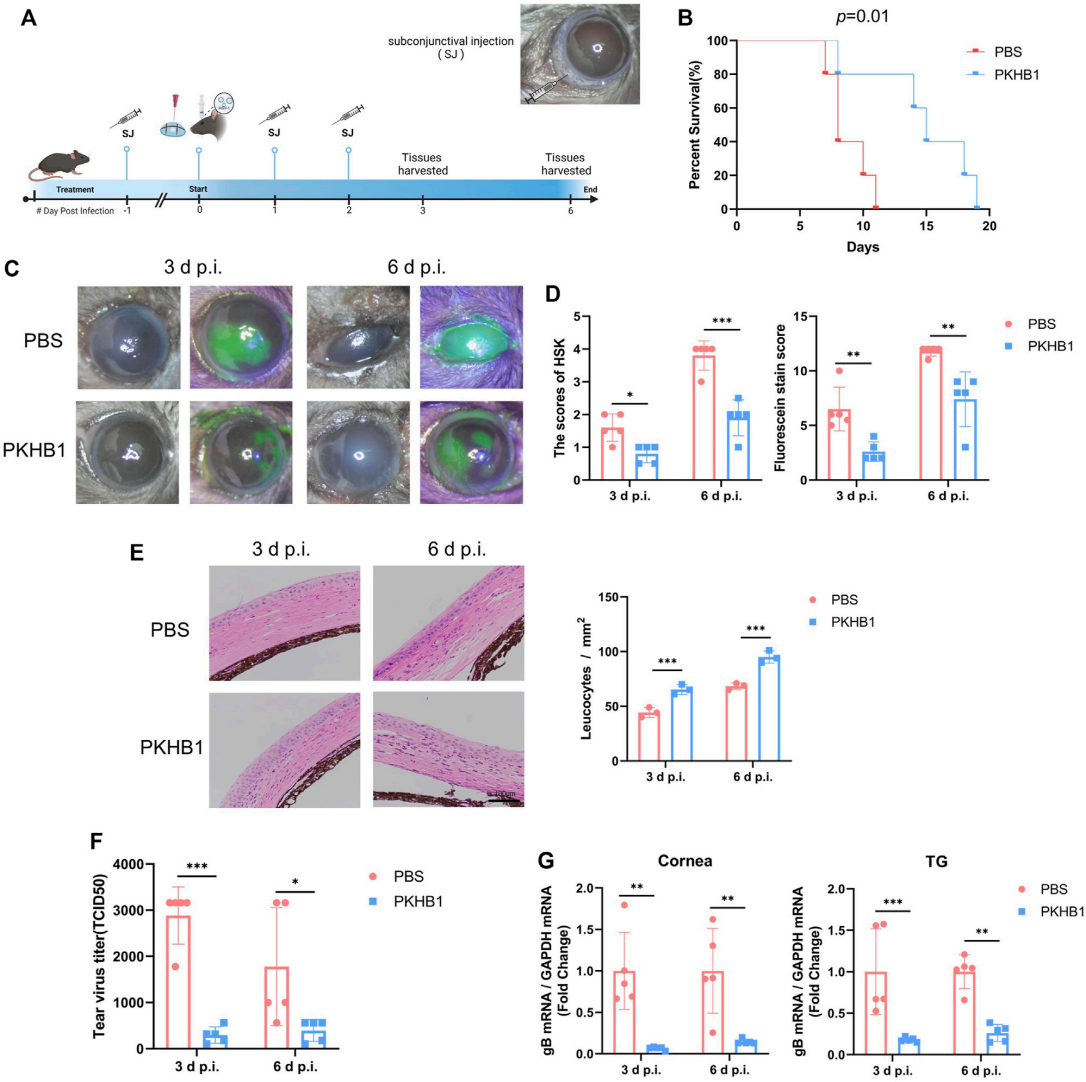
Subconjunctival injection of PKHB1 peptide inhibited HSV-1 replication and alleviated HSK lesions. **(A)** Drug delivery procedure and photograph of mice eyes after subconjunctival injection. **(B)** PKHB1-treated mice had longer survival rates. Photographs **(C)** and scores **(D)** of corneal lesions and sodium fluorescein staining in mice (*n* = 3–5). **(E)** HE staining of mouse corneal sections and quantifying leukocytes infiltration. **(F)** Virus titers of mouse tear swabs were calculated based on the Reed-Muench method. Virus titers of the PKHB1-treated mice were significantly lower (*n* = 5). **(G)** qRT-PCR was performed to detect the expression levels of HSV-1 gB in mice corneas and TGs at 3 d p.i. and 6 d p.i. (*n* = 5), which were significantly reduced in the PKHB1-treated mice. GAPDH was used as an internal reference. Data were presented as mean ± SD of three independent experiments. (*p < 0.05, **p < 0.01, ***p < 0.001)

We injected the peptide or PBS subconjunctivally 1 day before corneal HSV-1 infection. The subconjunctival injection of PKHB1 peptide did not cause ocular surface injury by observation before modeling (Supplementary Figure S2A). The infection was conducted unilaterally, and the contralateral eyes of all mice were free of epithelial lesions (Supplementary Figure S2B). After pre-treatment, the HSK model was constructed by the corneal scratch method, and the PKHB1 peptide or PBS was continuously injected for 2 days (Figure 2A). All mice in the PBS-treated group died within 11 d p.i. while PKHB1-treated mice lived until 19 d p.i. (Figure 2B). It suggested that PKHB1 peptide treatment significantly improved the survival rate of HSK mice compared to those treated with PBS. We chose the 3 d p.i. and 6 d p.i. to observe, photograph and collect samples. At 3 d p.i., the corneas of PBS-treated mice showed cloudiness and lamellar staining of sodium fluorescein, while the corneas of PKHB1-treated mice showed mild edema and dotted staining of sodium fluorescein (Figure 2C). The degree of HSK corneal lesions increased with the duration of infection. At 6 d p.i., PBS-treated mice presented a large amount of secretion around the eyelids leading to difficulty in eye-opening and obvious blepharitis. In contrast, PKHB1-treated mice showed less severe symptoms, with the cloudy portion and the sodium fluorescein staining involving only parts of the cornea instead of the entirety (Figure 2C; Supplementary Figure S2C). Using the HSK lesion degree score and sodium fluorescein staining score to quantify the symptoms, mice injected with PKHB1 peptide showed significant relief of clinical symptoms (Figure 2D). Corneal pathological changes were evaluated by HE staining, as shown in Figure 2E. By observing and quantifying leukocytes, we found that compared with PKHB1-treated mice, PBS-treated mice showed thickened corneal edema and a decrease in leukocytes infiltration.

To investigate the virus levels in the eyes of both groups, we added mice tear swabs to Vero cells and calculated TCID_50_ using the method of Reed-Muench. After injection of PKHB1 peptide, the virus levels in tears collected at 3 d p.i. and 6 d p.i. both decreased (Figure 2F). We further examined the expression of viral genes in the ocular tissues by qRT-PCR. The expressions of HSV-1 gB were significantly reduced in both the corneas and trigeminal ganglions (TGs) of PKHB1-treated mice at 3 d p.i. and 6 d p.i. (Figure 2G).

The results showed that subconjunctival PKHB1 peptide injection reduced HSV-1 levels in the eye and thus alleviated the severity of HSK.

### Topical PKHB1 peptide treatment increased the number of macrophages, especially the M1 type in herpes simplex keratitis *in vivo*

**FIGURE 3:**
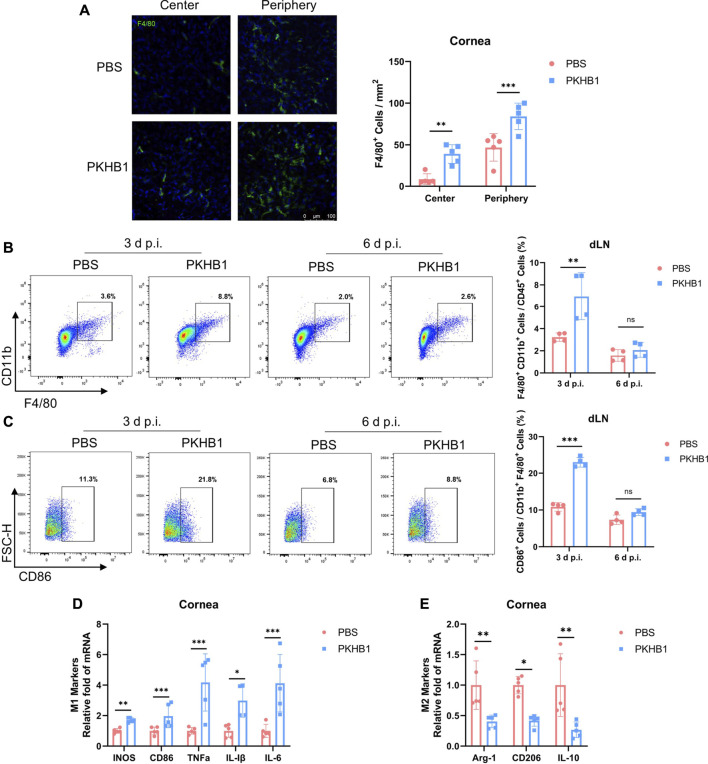
Subconjunctival injection of PKHB1 peptide increased the number of macrophages on the ocular surface and in the dLNs. **(A)** Representative micrographs (20 ×) of F4/80 (macrophages maker) expressing cells in the central and peripheral cornea (*n* = 5). Bar charts showing the density of macrophages in the cornea in each treatment group. Representative flow cytometry plots and bar chart showing the frequency of CD45^+^ CD11b^+^ F4/80^+^ cells **(B)** and CD86^+^ cells **(C)** in the dLNs of PKHB1 peptide or PBS-treated mice at 3 d p.i. and 6 d p.i. (*n* = 4). PKHB1 peptide increased the number of macrophages in the dLNs in the early stage of infection. The expression levels of genes for M1 **(D)** and M2 **(E)** macrophages polarizing cytokines in mice corneas at 3 d p.i. were measured by qRT-PCR (*n* = 5). GAPDH was used as an internal reference. Data were presented as mean ± SD of three independent experiments. (*p < 0.05, **p < 0.01, ***p < 0.001)

Macrophages played a crucial role in the pathogenesis of early HSK. Thus, we further investigated the effect of PKHB1 peptide on ocular macrophages. We collected corneas from mice at 3 d p.i. and performed immunofluorescence staining of the macrophage marker F4/80. We found that the PKHB1 peptide promoted macrophage infiltration in the peripheral and central cornea (Figure 3A). Following the gating strategy in Supplementary Figure S1A, we found that 99% of the cells in the dLNs single-cell suspensions were live cells. By flow cytometry according to the gating strategies in Supplementary Figure S1B, we found that PKHB1 peptide increased the percentage of macrophages, predominantly M1 types in the dLNs at 3 d p.i. but did not affect macrophages later at 6 d p.i. (Figures 3B,C). As shown in the figure, the gene expression levels of M1 macrophage polarizing cytokines such as inducible nitric oxide synthase (iNOS), CD86, tumor necrosis factor α (TNF-α), interleukin 1β (IL-1β), and interleukin 6 (IL-6) were significantly higher in the PKHB1-treated mice corneas compared with PBS-treated mice at 3 d p.i. (Figure 3D). In comparison, the transcriptional levels of M2 macrophage polarizing cytokines such as arginase 1 (Arg-1), CD206, and interleukin 10 (IL-10) were significantly lower at 3 d p.i. (Figure 3E).

These results suggest that local injection of PKHB1 peptide increased the number of macrophages in the corneas and dLNs of HSK mice in the early infection period, which was dominated by M1 classic macrophages with powerful antiviral function.

### Topical PKHB1 peptide treatment increased the number of dendritic cells in herpes simplex keratitis *in vivo*

**FIGURE 4:**
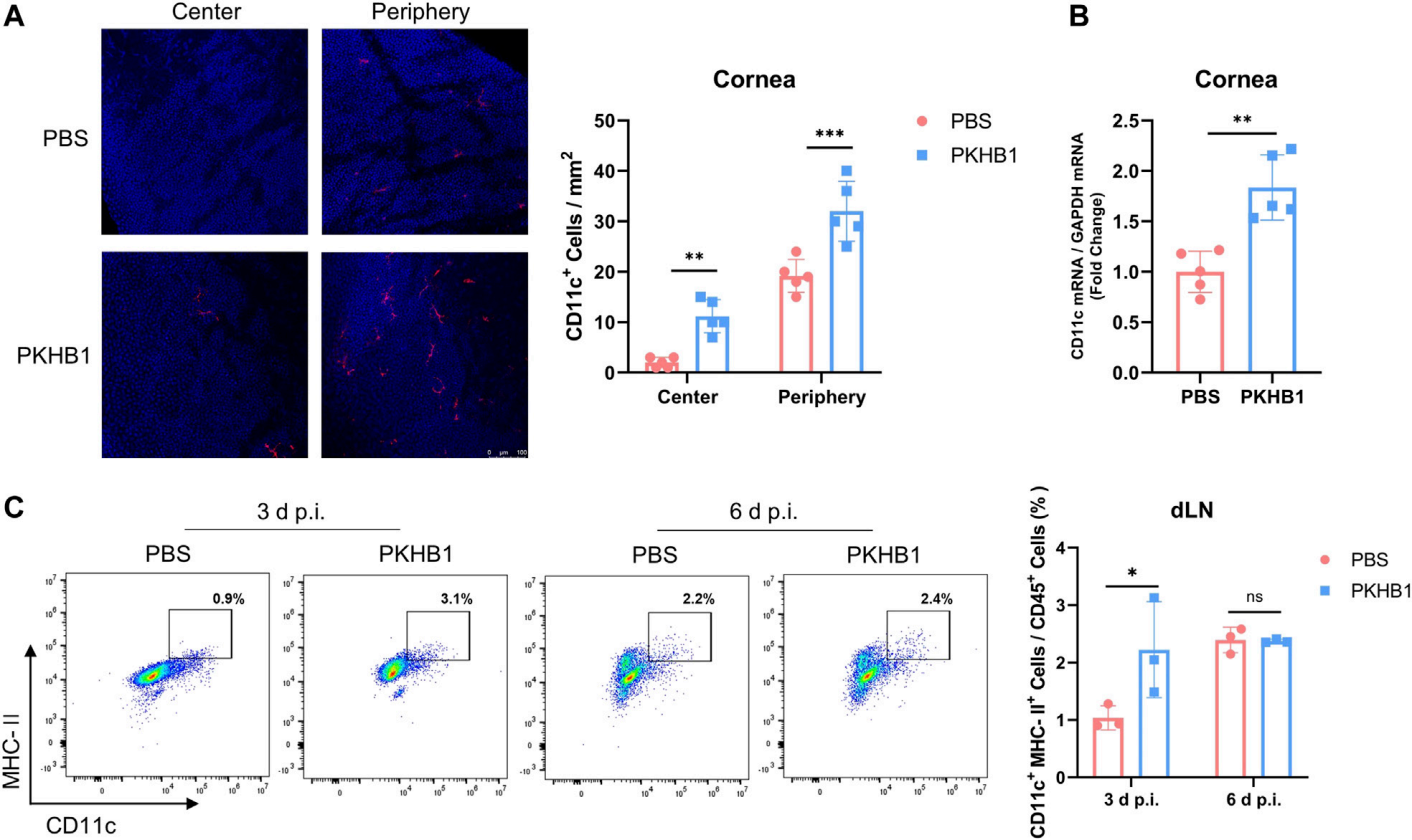
Subconjunctival injection of PKHB1 peptide increased the number of DCs on the ocular surface and in the dLNs. Representative micrographs and bar charts **(A)** of cells expressing CD11c (dendritic cell maker) in the central and peripheral corneas after different treatments (20 ×) (*n* = 5). **(B)** Detection of transcriptional levels of mouse corneal CD11c by qRT-PCR with GAPDH as an internal reference (*n* = 5). Representative flow cytometry plots and bar charts **(C)** showing the frequency of CD45^+^ CD11c^+^ MHC- II^+^ DCs in dLNs of PKHB1 peptide or PBS topically treated HSK mice at 3 d p.i. and 6 d p.i. (*n* = 3). PKHB1 peptide increased the number of DCs in the dLNs in the early stages of infection. Data were presented as mean ± SD of three independent experiments. (*p < 0.05, **p < 0.01, ***p < 0.001)

We further explored whether there were changes in DCs, the powerful APCs, after PKHB1 peptide administration. Immunofluorescence staining showed that corneas from PKHB1-treated mice had more DCs in both the central and the peripheral cornea than PBS-treated mice at 3 d p.i. (Figure 4A). The qRT-PCR assay of transcriptional levels of CD11c in the corneas was consistent with the trend (Figure 4B). Flow cytometry data according to the gating strategies in Supplementary Figure S1C showed higher frequencies of CD11c^+^ MHC-II^+^ DCs in the dLNs of PKHB1-treated mice in the early stages of infection compared to PBS-treated mice (Figure 4C).

### Topical PKHB1 peptide treatment increased the number of CD8^+^ T cells in the draining lymph nodes

**FIGURE 5:**
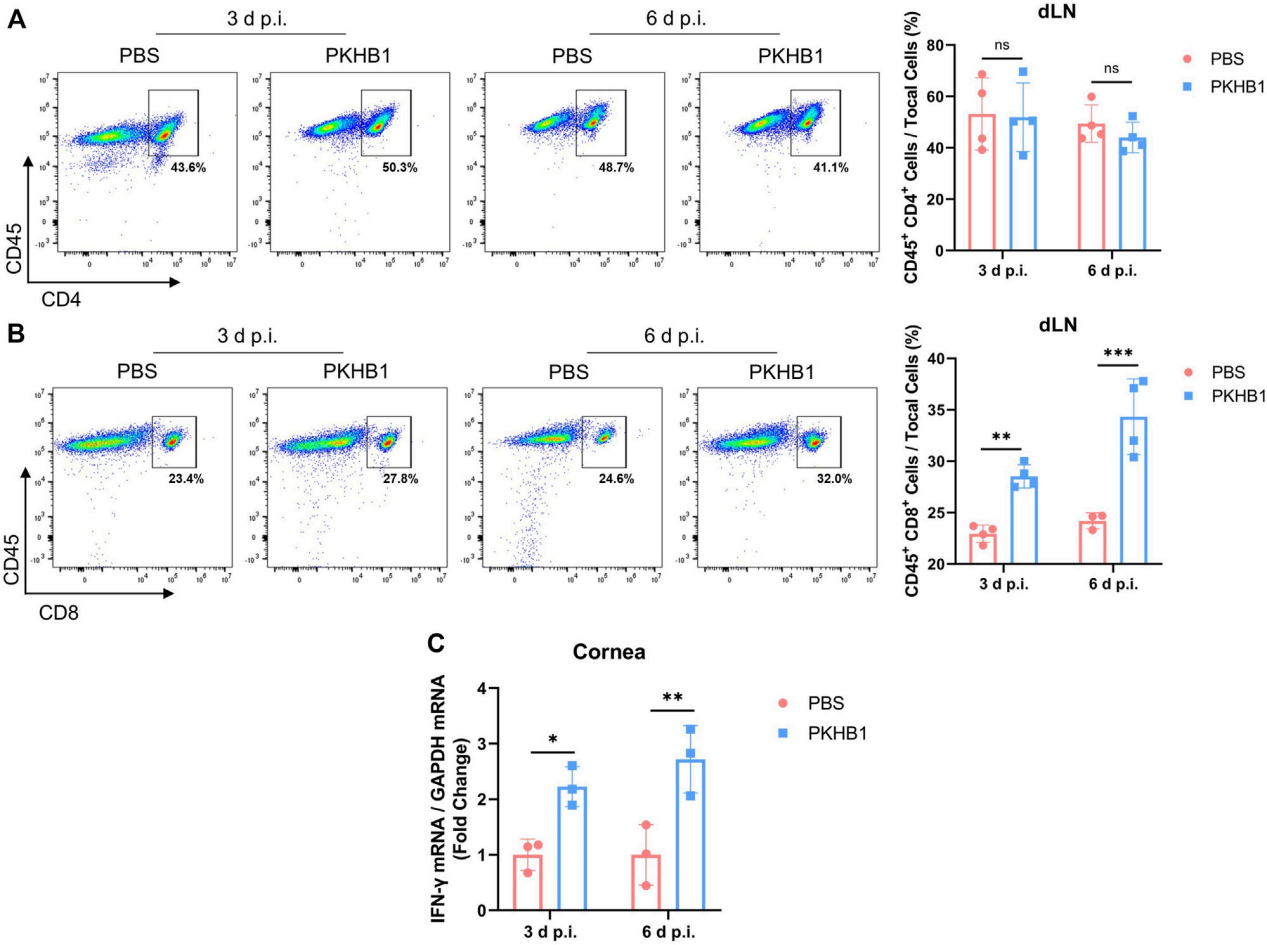
Subconjunctival injection of PKHB1 peptide increased the number of CD8^+^ T cells in the dLNs. Representative flow cytometry plots and bar charts showing the frequency of CD4^+^ T cells **(A)** and CD8^+^ T cells **(B)** in the dLNs of PKHB1 peptide or PBS topically treated HSK mice at 3 d p.i. and 6 d p.i. (*n* = 4). There was no change in the number of CD4^+^ T cells but a significant increase in the number of CD8^+^ T cells after PKHB1 peptide administrations. **(C)** qRT-PCR detection of changes in the expression levels of IFN-γ in the mice corneas (*n* = 3). The increased expression once again demonstrated that the PKHB1 peptide activated the immune response, leading to an increase in the ability of the cornea to fight viruses. Data were presented as mean ± SD of three independent experiments. (*p < 0.05, **p < 0.01, ***p < 0.001)

To investigate whether APCs that have phagocytized the pathogens present viral antigens to the dLNs and activate T lymphocytes to elicit downstream anti-viral immune responses, we obtained dLNs from HSK mice at 3 d p.i. and 6 d p.i. and detected the frequencies of CD4^+^ T cells and CD8^+^ T cells by flow cytometry according to the gating strategies in Supplementary Figure S1D. There was no change in the number of CD4^+^ T cells, but the number of CD8^+^ T cells in the dLNs increased significantly after PKHB1 peptide injection (Figures 5A,B). The increased expression of IFN-γ on infected corneas also indicated that the PKHB1 peptide activated the anti-viral immune response, enhancing the ability of the cornea to fight virus infections. (Figure 5C).

### PKHB1 peptide may enhance the local immune response by promoting the occurrence of immunogenic cell death

According to previous studies, PKHB1 peptide has been shown to induce ICD in various oncological diseases such as breast cancer and leukemia. We further investigated the specific mechanism underlying its protective role in HSK pathogenesis, such as enhancing immune response to inhibit viral replication and alleviate ocular symptoms.

**FIGURE 6:**
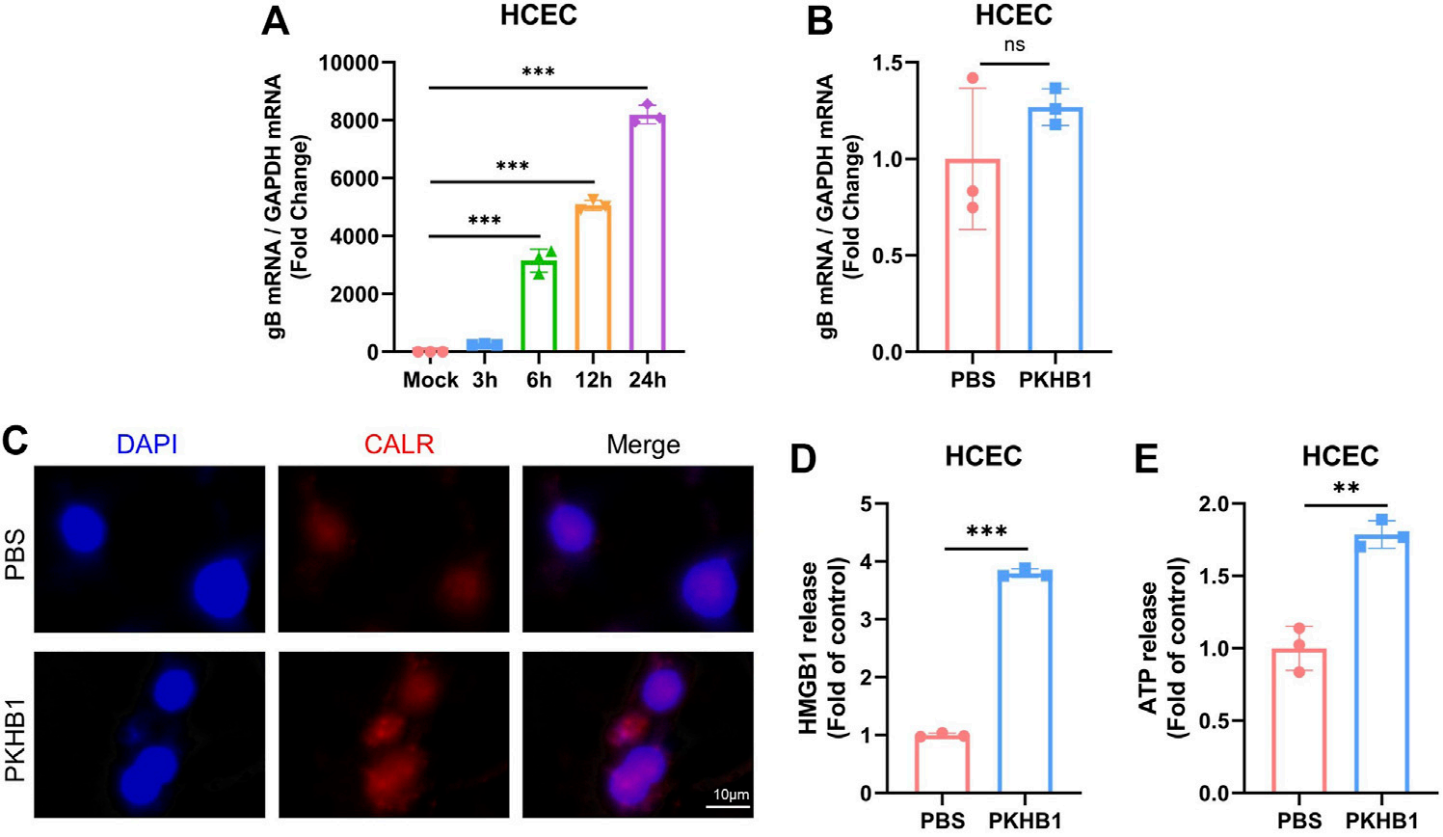
PKHB1 peptide may enhance the local immune response by promoting the occurrence of ICD in infected cells. **(A)** HSK *in vitro* infection model in HCECs. **(B)** Infected HCECs were treated with PBS or PKHB1 peptide and the expression levels of HSV-1 gB were measured using qRT-PCR. GAPDH was used as an internal reference. PKHB1 peptide did not directly inhibit HSV-1 replication, but increased the release of more immunostimulatory factors from infected cells. **(C)** CALR exposure was observed by microscopy in HCECs using CALR staining and DAPI. Representative graphs of HMGB1 **(D)** or ATP **(E)** release in the extracellular supernatants of PKHB1 or PBS-treated cells (*n* = 3). Data were presented as mean ± SD of three independent experiments. (*p < 0.05, **p < 0.01, ***p < 0.001)

HCECs were cultured *in vitro* and infected with HSV-1 (MOI = 1) (Figure 6A). The incubation of HCECs alone with PKHB1 peptide did not alleviate the replication of the virus without the co-culture of HCECs with immune cells (Figure 6B). We hypothesized that PKHB1 could induce the release and exposure of principal DAMPs in infected cells to activate immune cells for downstream immune responses. On the one hand, immunofluorescence microscopy confirmed the exposure of CALR (one of the essential DAMPs associated with ICD) induced by PKHB1 peptide treatment (Figure 6C). Secondary controls were incubated by fluorescent secondary antibody and DAPI without CALR antibody staining (Supplementary Figure S3). On the other hand, the release of HMGB1 and ATP were assessed in the supernatants of infected cells. Results showed a significant release of HMGB1 and ATP in the supernatants of PKHB1-treated cells compared with PBS-treated cells (Figures 6D,E).

## Discussion

**FIGURE 7:**
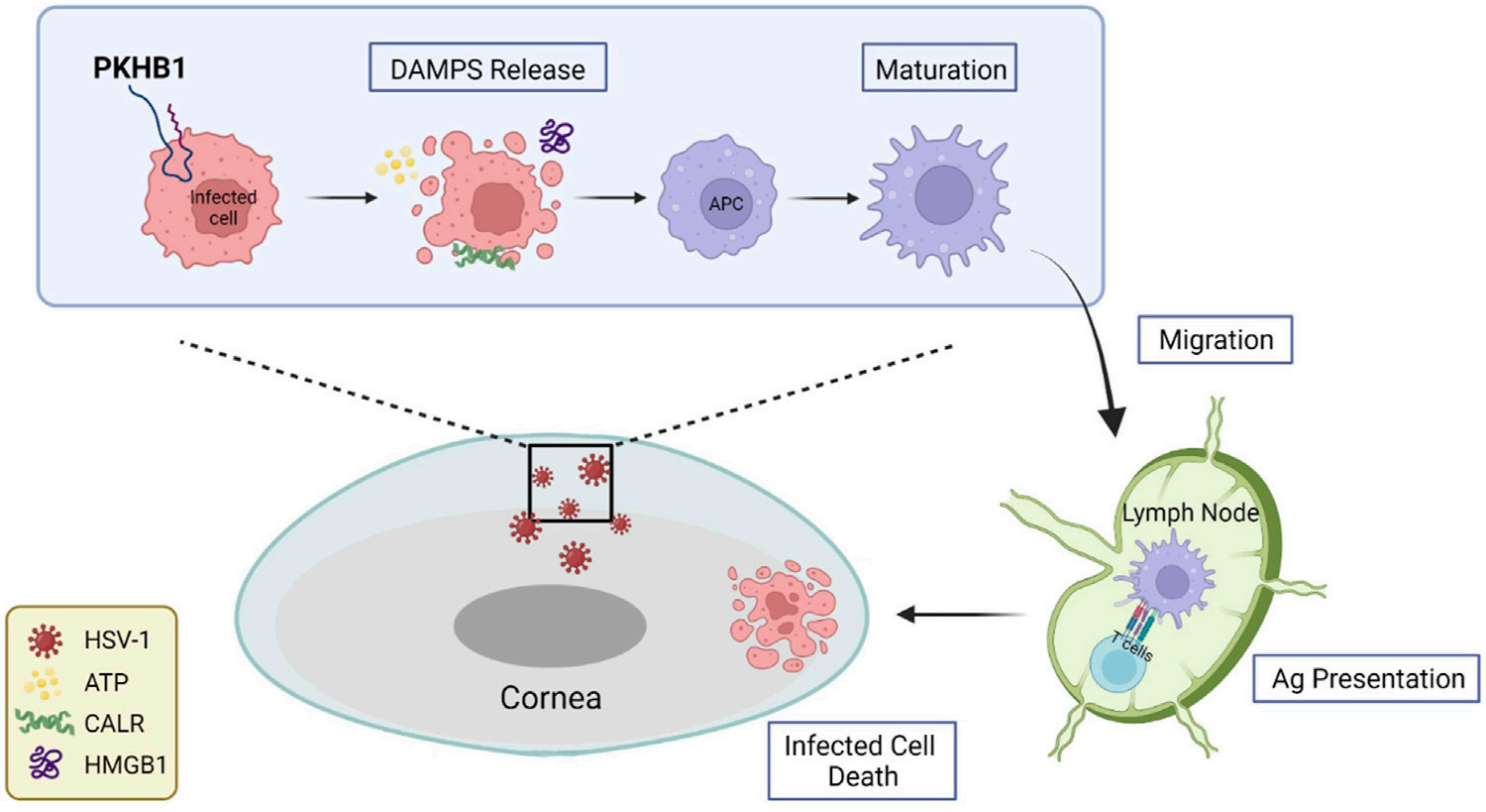
Schematic representation of PKHB1-mediated ICD. PKHB1 peptide promoted the recruitment and maturation of APCs by inducing the release of large amounts of DAMPs from infected cells. APCs that phagocytized antigenic materials transferred to the dLNs and triggered cytotoxic T lymphocyte-dependent immune responses, ultimately killing infected cells to alleviate HSK.

After HSV-1 invades the cornea, evasion of host immunity leads to rapid HSV-1 replication and damage to the epithelium, with patients experiencing pain, photophobia, blurred vision, tearing and redness. Viruses are not cleared in the time leading to repeated episodes of HSK, and the patients develop progressive and irreversible corneal scarring and eventually blindness. In this study, we investigated the role of PKHB1 peptide, an ICD inducer capable of activating tumor immune responses, in HSK pathogenesis. This is the first study to explore the role of PKHB1 peptide in infectious diseases. We administered PKHB1 peptide subconjunctivally to HSK mice and discovered that the ocular surface symptoms and survival rates of the PKHB1-treated mice were significantly improved, and HSV-1 levels in the corneas and TGs were significantly decreased. Then, we found an increased number of infiltrating APCs, including macrophages and DCs, in the corneas and dLNs. We also verified the increased percentage of CD8^+^ T cells in the dLNs. *In vitro* experiments further confirmed that PKHB1 peptide functioned as an ICD inducer in HSK (Figure 7).

The subconjunctival injection can increase the concentration and prolong the duration of action of the drug on the ocular surface (Rafiei et al., 2020), so we chose subconjunctival injection as the delivery method of PKHB1 peptide in HSK mice. In the PKHB1-treated mice, the clinical symptoms were significantly relieved, the degrees of lesions and the points of corneal sodium fluorescein staining scores were reduced, and the survival was significantly prolonged. Reduced HSV-1 levels were observed in both the tear fluid and the corneas of PKHB1-treated mice (Figure 2). HSV-1 is known to invade the corneal nerves and travel retrogradely through the axon to the TG to establish latency (Wang et al., 2022). When the host’s immunity weakens, activation of the latent HSV-1 causes the recurrence of HSK (Jamali et al., 2020). The transcript level of HSV-1 gB was decreased in the TGs of PKHB1-treated mice (Figure 2), suggesting that PKHB1 peptide not only reduced viral replication in primary HSK, but may also play a protective role in recurrent HSK.

As the first immune cells to come into contact with the virus, macrophages can rapidly migrate to infected tissues, engulf the viruses and infected cells, and process their antigens to present signals to other immune cells such as T cells, which in turn activate downstream immune responses and exert anti-infective effects (Xu et al., 2019). In PKHB1-treated mice, macrophages in both the corneas and dLNs were significantly increased, suggesting that PKHB1 peptide may reduce viral replication by regulating macrophages (Figure 3). It has been found that M1 macrophages secrete various cytokines that play a key role in clearing virus replication in the early stage of HSK (Jaggi et al., 2021). So, we also assayed the macrophages polarizing cytokines. We found that gene expression levels of M1 macrophage polarizing cytokines such as iNOS, CD86, TNF-α, IL-1β, and IL-6 were significantly higher in the PKHB1-treated mice corneas compared with PBS-treated mice. In comparison, the transcriptional levels of M2 macrophage polarizing cytokines such as Arg-1, CD206, and IL-10 were significantly lower (Figure 3). These also further suggest an anti-infective role for M1 macrophages in HSK. Also, our study showed that the number of DCs in the corneas and dLNs of PKHB1-treated mice also increased significantly, indicating that macrophages may team up with DCs to present antigens and initiate an adaptive immune response (Figure 4).

Successful binding of naive CD8^+^ T cells with APCs stimulates immature CD8^+^ T cells to become activated CD8^+^ T cells with cytotoxic functions (Castellino and Germain, 2006). CD8^+^ T cells secrete cytotoxic proteins such as perforin and granzyme to kill target cells directly while also killing them indirectly by releasing IFN-γ (Chávez-Galán et al., 2009). Through our study, we found that the number of CD8^+^ T cells were significantly increased in the dLNs of PKHB1-treated mice at 6 d p.i. (Figure 5), while APCs (macrophages and DCs) in the dLNs were significantly increased at 3 d p.i. (Figures 3, 4). It may suggest that PKHB1 peptide facilitated the presentation of antigen to dLNs by APCs that have phagocytized the pathogens, activating T lymphocytes to elicit downstream antiviral immune responses and kill infected target cells to clear the foci of infection. PKHB1 peptide increased the secretion of IFN-γ by immune cells enhancing the ability of the cornea to fight virus infections (Figure 5). A variety of immune cells can secrete IFN-γ, such as macrophages, natural killer cells, and T cells. In a follow-up study, which immune cell is the one that plays a key role in the increased IFN-γ secretion will be further explored.

PKHB1 peptide has been shown to induce ICD in various oncological diseases such as breast cancer and leukemia (Uscanga-Palomeque et al., 2019; Calvillo-Rodríguez et al., 2022). The low immunogenicity of infected cells is a significant obstacle to anti-infective therapy. One way to reactivate an effective anti-infective immune response is through the release of DAMPs, which can be achieved by ICD. PKHB1 peptide induced CALR exposure, HMGB1 and ATP release (Figure 6), which may promote the uptake of dying cells as well as the recruitment, maturation, and cross-presentation activity of APCs. PKHB1 peptide did not affect the infection of the cells *in vitro* in an environment without immune cell co-culture (Figure 6), which again indicated that immune cells might play an irreplaceable role in the alleviation of HSK by PKHB1 peptide. In the future study, HCECs could be co-culture with immune cells such as macrophages and DCs further to demonstrate the therapeutic effect of PKHB1 peptide *in vitro*.

However, the most critical DAMP for the role of PKHB1 peptide in HSK and the specific mechanism of its immune enhancement has yet to be established. It is possible to use different types of agonists and inhibitors of DAMPs and compare the difference in efficacy between them and PKHB1 to determine the DAMP that plays the most critical role. The relationship between the release of DAMPs and PKHB1 concentration can be observed by adding different concentrations of PKHB1 to the infected cells. During the process of HSK, an inadequate immune response increases disease susceptibility, whereas an overreaction induces corneal clouding through inflammatory cell damage; this indicates that balanced immunopathological responses are crucial for preventing corneal clouding, scar formation and permanent vision loss (Jaggi et al., 2020). Thus, controlling the dose and frequency of the peptide to maintain the balance between antiviral capacity and inflammatory response is of great significance. The optimal time for PKHB1 peptide administration can be explored by setting different timing groups, such as administration before infection, administration while infecting, and administration after the appearance of symptoms. These need to be followed up with more in-depth studies.

There is an urgent need to develop new approaches for treating HSK due to reasons such as drug resistance or side effects of existing drugs. In this study, we explored the role and mechanism of PKHB1 peptide, an ICD inducer, in HSK. Our findings indicated that PKHB1 peptide increased the immunogenicity of infected cells by inducing the release of large amounts of DAMPs and promoting the recruitment and maturation of APCs, thereby triggering a cytotoxic T lymphocyte-dependent immune response that ultimately treated HSK. A variety of viruses are known to evade host immune recognition, block immune signaling pathways and eventually achieve suppression and escape of immunity when infecting different tissues, such as human immunodeficiency virus (van Kooyk et al., 2003), hepatitis virus (Rosenberg, 1999), dengue virus (Uno and Ross, 2018), etc. And PKHB1 peptide, as an ICD agonist, can activate the immune response by inducing infected cells to release more DAMPs to fight against immune evasion. So, this paper not only provided new ideas for treating HSK by immunotherapy and targeted therapies, but also offered a more theoretical basis for applying PKHB1 peptide in other infectious diseases.

## Data Availability

The original contributions presented in the study are included in the article/Supplementary Material, further inquiries can be directed to the corresponding author.

## Supplementary material

SUPPLEMENTARY FIGURE S1 Flow cytometry gating strategies. **(A)** Gating strategies for Live/Dead cells. **(B)** Gating strategies for CD45^+^ Live lymphocytes expressing F4/80, CD11b and CD86 were gated to analyze macrophages percentage. **(C)** Gating strategies for CD45^+^ Live lymphocytes expressing CD11c and MHC- II were gated to analyze DCs percentage. **(D)** Gating strategies for CD45^+^ Live lymphocytes expressing CD4 and CD8 were gated to analyze T cells percentage. **(E)** Gating strategies for isotype control.

SUPPLEMENTARY FIGURE S2 Mice eyes images. **(A)** Images taken after sub-conjunctival injection and before infection. **(B)** Contralateral eye images. **(C)** Fluorescein staining images of all animals.

SUPPLEMENTARY FIGURE S3 Cell fluorescence images. Infected HCECs were incubated by fluorescent secondary antibody and DAPI without CALR antibody staining.
