# Supplementary figures and images for: PKHB1 peptide induces antiviral effects through induction of immunogenic cell death in herpes simplex keratitis
Source: Front Pharmacol. 2022 Dec 1;13:1048978. doi: 10.3389/fphar.2022.1048978 (PMC9751201; doi:10.3389/fphar.2022.1048978)

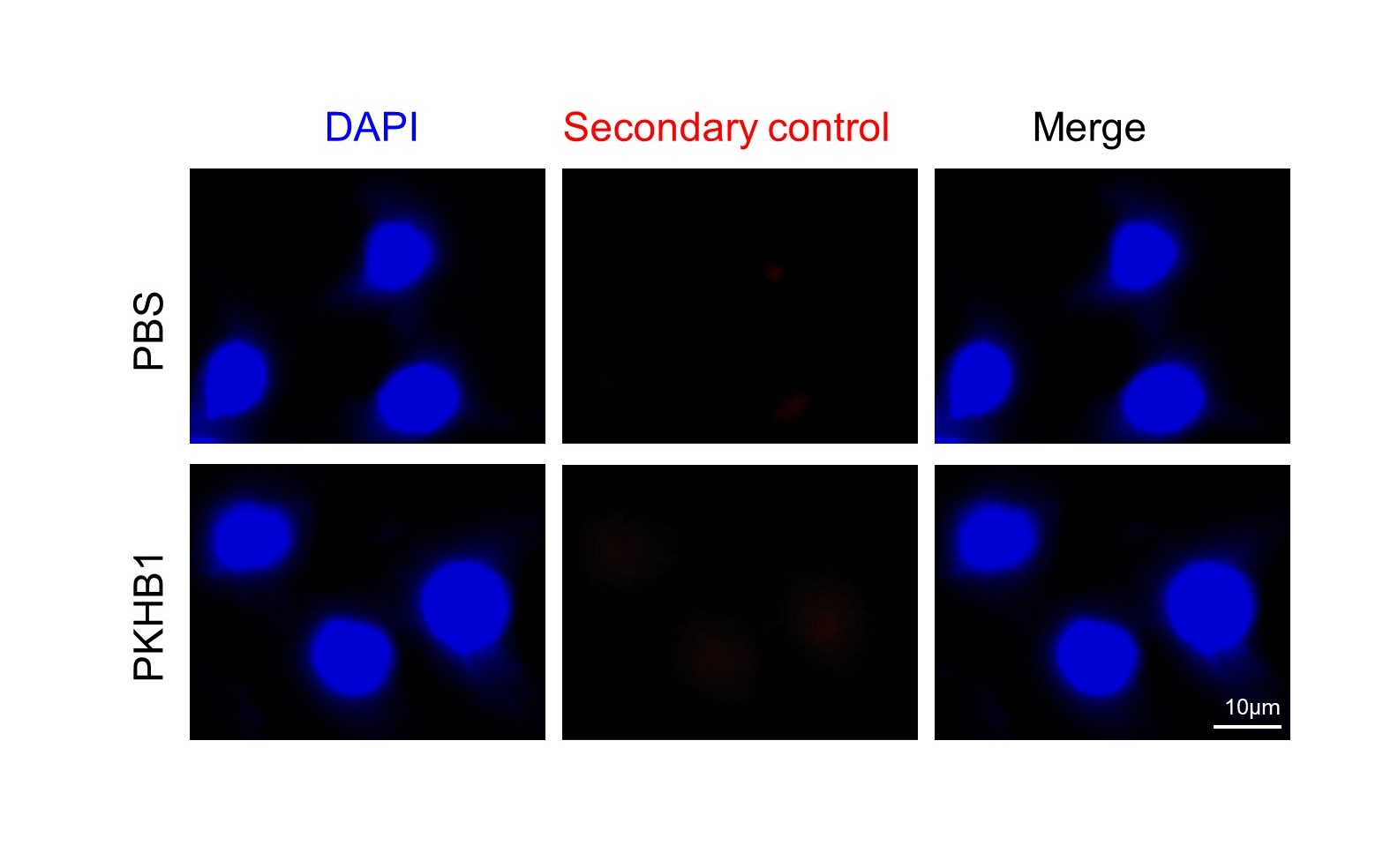

Supplement: Supplementary file 1 [file Image3.JPEG]

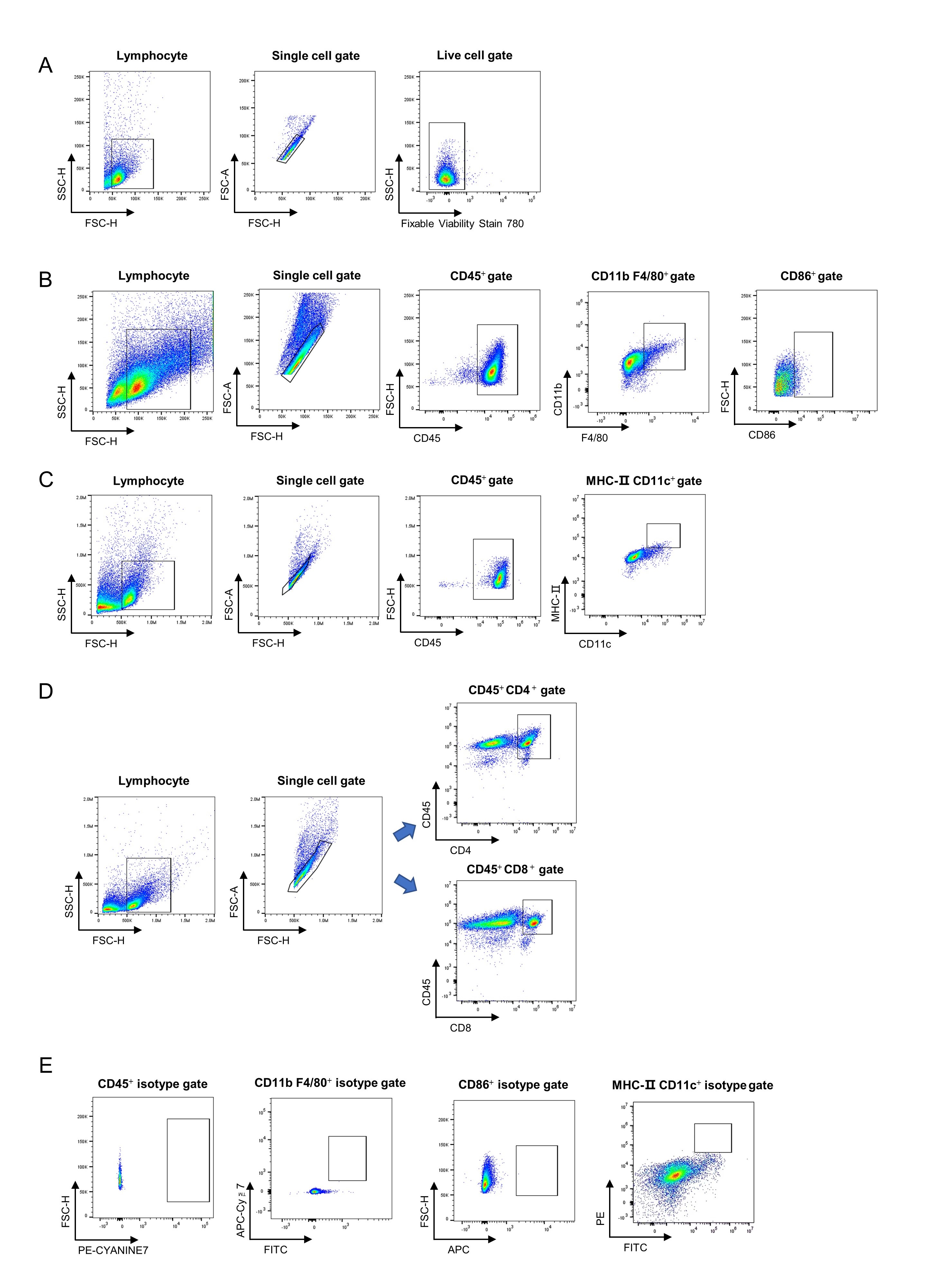

Supplement: Supplementary file 2 [file Image1.JPEG]

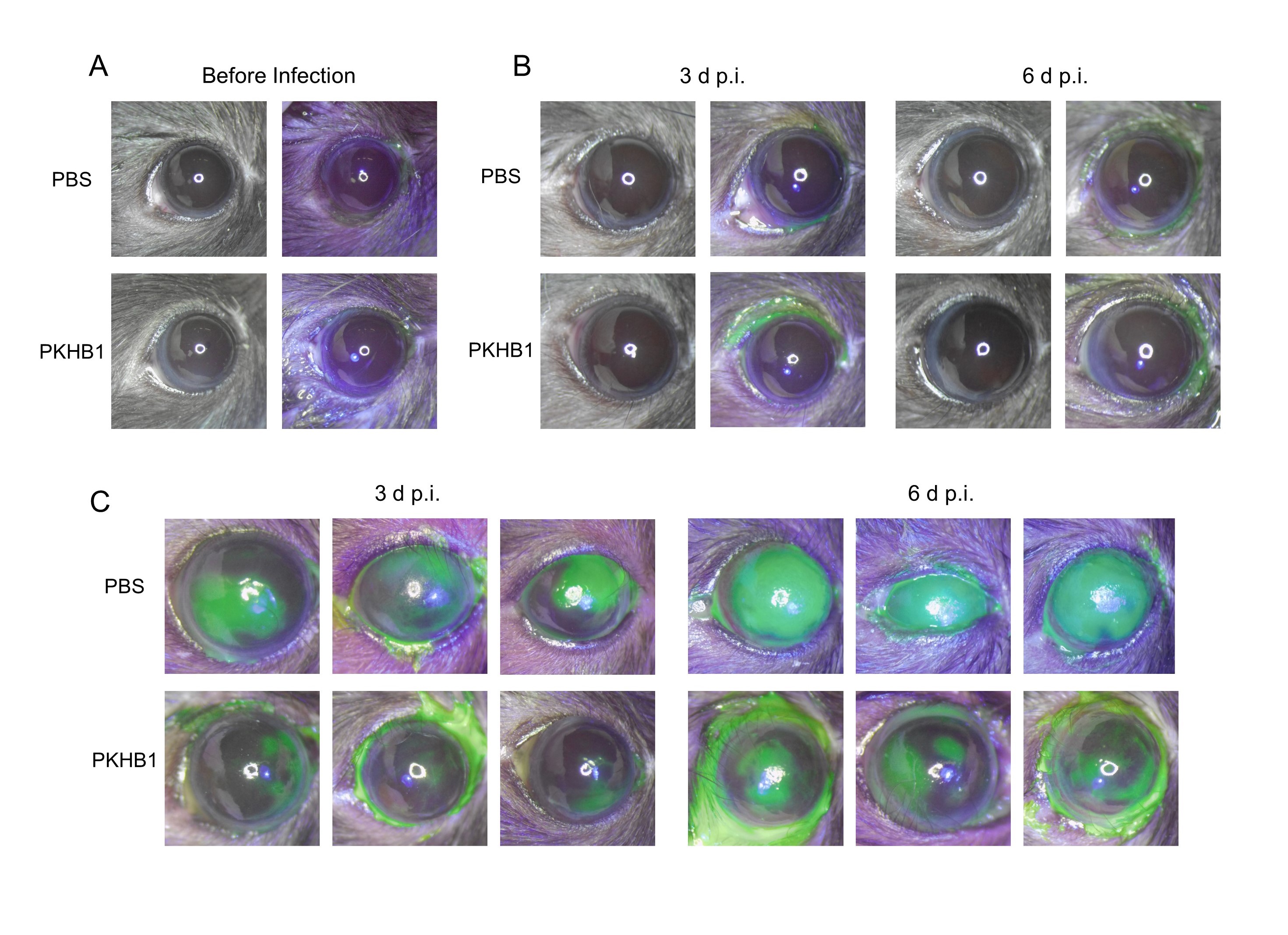

Supplement: Supplementary file 3 [file Image2.JPEG]
